# Power of Hypoperfusion in Predicting Recurrent Transient Ischemic Attacks: Protocol of a Prospective Cohort Study

**DOI:** 10.3389/fnhum.2021.654383

**Published:** 2021-06-24

**Authors:** Yue Wang, Huazheng Liang, Lingjing Jin, Shaoshi Wang

**Affiliations:** ^1^Department of Neurology, Shanghai Fourth People's Hospital Affiliated to Tongji University School of Medicine, Shanghai, China; ^2^Department of Neurology, Translational Research Institute of Brain and Brain-Like Intelligence, Shanghai Fourth People's Hospital Affiliated to Tongji University School of Medicine, Shanghai, China; ^3^Department of Neurology, Tongji Hospital, Tongji University School of Medicine, Shanghai, China; ^4^Department of Neurological Rehabilitation, Shanghai Yangzhi Rehabilitation Hospital (Shanghai Sunshine Rehabilitation Center), Tongji University School of Medicine, Shanghai, China

**Keywords:** transient ischemic attack, hypoperfusion, magnetic resonance perfusion, prospective, cohort, recurrence

## Abstract

**Background:** Transient ischemic attack (TIA) has a high incidence of recurrent vascular events. Hypoperfusion is one of the factors that are closely correlated with 7-day recurrence of TIA. This study aimed to evaluate the power of hypoperfusion shown on magnetic resonance (MR) perfusion imaging in predicting the incidence of 7-day recurrence of ischemic events after TIA.

**Methods/Design:** REATTACK is a prospective multi-centered cohort study on the correlation between MR perfusion and TIA recurrence. Ninety patients aged ≥18 years with recent (<7 days after onset) clinical TIA will be continuously included. All the patients will undergo diffusion-weighted imaging (DWI) and perfusion-weighted imaging (PWI) assessments within 24 h after the onset of TIA. The subjects will then be divided into a PWI positive group and a PWI negative group according to the time-to-maximum of the residue function (*T*_*max*_). PWI will be repeated after 7 days and in 3 months. The primary clinical outcome will be the recurrence of TIA within 7 days after the onset of TIA. Secondary outcomes will be the recurrence of TIA in 3 months and modified Rankin scale (mRS) score. A chi-square test will be performed to compare the difference in the incidence of recurrent TIA between the two groups, and rank sum test in the mRS score. Multivariate logistic regression will be simultaneously performed to analyze the risk factors for the recurrence of TIA.

**Discussion:** The results of this study will confirm whether abnormal *T*_*max*_ helps to identify the patients with TIA who have high risks of recurrent ischemic events. This would largely improve the prognosis of patients with TIA.

**Trial Registration:** www.chictr.org.cn, registration number: ChiCTR2000031863, registered on 12 April 2020.

## Background

Transient ischemic attack (TIA) has been defined as a short-lasting episode of neurological dysfunction without evidence of acute infarction (Easton et al., 2009). Approximately 24 million people experience TIA every year in China (Wang et al., 2015). Patients with recent TIAs are at a high risk for recurrent strokes or other vascular events (Lavallee et al., 2007; Amarenco et al., 2016). For example, patients with TIA have a 4–10% possibility of developing a stroke within 7 days and a 10–20% (average 11%) possibility within 90 days, much higher than the risk of developing recurrent strokes (2–7%, average 4%) within 90 days after an acute ischemic stroke (Kelly et al., 2016). Urgent diagnosis and treatment can significantly reduce the risk of recurrent ischemic events after TIA (Amarenco et al., 2016; Kim et al., 2016). A cohort study in the UK showed that optimal secondary prevention decreased the incidence of recurrent ischemic events after TIA from 10.3 to 2.1% (Rothwell et al., 2007).

A recent study raised the hypothesis that patients with TIA have different degrees of recurrence, depending on clinical symptoms, pathological mechanisms, and treatments (Kelly et al., 2016). How to identify TIA patients at high risk of recurrence and provide timely treatment is a problem that needs to be solved urgently in the clinic. The age, blood pressure, clinical symptoms, duration, and diabetes score (ABCD2, 0–7 points) is a stratified approach based on clinical manifestations. It aims to facilitate the evaluation of patients with stroke/TIA by non-neurology medical staff in the emergency room. For neurology professionals, its specificity is low (Amarenco et al., 2009; Giles and Rothwell, 2010; Wardlaw et al., 2015).

The morphological definition of TIA, that is, diffusion-weighted imaging (DWI)-negative TIA, did not clearly distinguish the tissue perfusion status of all patients with TIA. Low perfusion may be one of the mechanisms of TIA recurrence. Perfusion weighted imaging (PWI) is positive in 20–40% of patients with clinical TIA, while DWI is negative (Mlynash et al., 2009; Kleinman et al., 2012; Asdaghi et al., 2013; Grams et al., 2016; Lee et al., 2017). Previous studies have suggested that hypoperfusion might be associated with recurrent TIAs, stroke, and continued deterioration of neurological symptoms (Olivot et al., 2008; Asdaghi et al., 2011, 2013; Lee et al., 2017). The previous study conducted by the authors has assessed 66 patients with TIA with anterior circulation symptoms and found that hypoperfusion is present in 50% of the patients (33/66), with 31.8% (21/66) showing PWI-positive and DWI-negative findings (Wang et al., 2019b). In order to further explore whether the recurrence of TIA in patients with no lesions shown on DWI is related to hypoperfusion, 46 patients with normal DWI were followed up for 3 months in the study. The incidence of recurrent TIA was 41.2% (14/34) in the hypoperfusion group and 16.7% (2/12) in the normal perfusion group. There was a significant statistical difference between the two groups.

There are many modalities for assessing hypoperfusion. Although mean transit time (MTT) and time to peak (TTP) can be used to observe abnormal changes in perfusion in patients with TIA (Kleinman et al., 2012), time-to-maximum of residue function (*T*_*max*_) ≥ 4 s is currently the best one for evaluating perfusion abnormalities in the early stage (Olivot et al., 2009; Wang et al., 2019a,b). The previous results showed that the prevalence of *T*_*max*_ ≥ 4 s > 10 ml was 50% (33/66). Limb fatigue combined with *T*_*max*_ ≥ 4 s > 10 ml was an independent risk factor for recurrence (adjusted OR = 3.74, 95% CI = 1.02–13.64, *P* = 0.046). Therefore, it is feasible and clinically significant to use *T*_*max*_ ≥ 4 s to evaluate the perfusion status of patients with TIA.

The Prospective Cohort Study of Hypoperfusion in Predicting Recurrent Transient Ischemic Attacks (REATTACK) trial is, therefore, designed to explore the correlation between hypoperfusion and TIA recurrence as well as prognosis. By completing this prospective cohort study of patients with TIA with low perfusion (*T*_*max*_ ≥ 4 s > 10 ml), it is likely to further the insight into the pathological mechanism of TIA recurrence.

## Methods

### Study Design

REATTACK is a prospective cohort study with a 3-month follow-up period. The participants are patients who had TIA within 7 days of onset diagnosed by two neurologists. Brain DWI and PWI assessments will be completed within 24 h of admission, and *T*_*max*_ > 4 s > 10 ml is used to determine the presence of hypoperfusion. According to baseline MRI-PWI examination, the patients are divided into the PWI positive and PWI negative groups. DWI, PWI, fluid-attenuated inversion recovery (FLAIR), and magnetic resonance angiography (MRA) examinations will be repeated 7 days later. The main outcomes are the incidence of recurrent TIA within 7 days and in 3 months, and the mRS score in 3 months.

### Objectives

#### Primary Objective

▪   To assess the added diagnostic value of hypoperfusion demonstrated on PWI in predicting recurrent vascular events of patients with TIA within 7 days.

#### Secondary Objective

▪   To determine the prevalence and course of hypoperfusion and its effect on recurrent vascular events in patients with TIA 3 months after onset.

### Study Population and Setting

All patients will be recruited from the outpatient of the Neurology Department of Shanghai Fourth People's Hospital and Tongji Hospital; both are affiliated to Tongji University School of Medicine. TIA will be diagnosed by two experienced attending physicians in the Neurology Department.

### Inclusion Criteria

- Aged 18 years or older
- No intracerebral hemorrhage
- Patients or their relatives are willing to participate in the study and provide written informed consent
- Meet the diagnostic criteria for transient ischemic attack
- Present within 7 days of onset.

### Exclusion Criteria

- The recent episode lasted more than 1 h but <4.5 h upon arrival at the hospital
- Pre-stroke mRS ≥ 2
- History of severe bleeding or major surgery within 30 days
- Concomitant terminal illness with a life expectancy ≤12 months
- Any known hereditary or acquired hemorrhagic diathesis, coagulation factor deficiency, or intake of oral anticoagulants with INR > 1.7
- Platelet count <100,000/mm^3^
- Hemoglobin <10 g/dl
- Serum glucose level ≤50 mg/dl
- Severe renal insufficiency with creatinine clearance rate <30 ml/min
- Patients or their relatives refuse to or not available for MRI perfusion test
- Uncooperative patients in 3-month follow-up.

### Recruitment and Consent

Patients suspected of clinical TIA will be recruited by attending general practitioners or neurologists at the time they present to the neurology clinic. They and/or their relatives will be asked whether they agree to be contacted by researchers regarding the possibility of participating in this study. If the patients and/or their relatives agree to do so, their eligibility will be assessed, and the detailed procedures of this study will be explained to them. Lastly, written informed consent will be obtained from them if they are eligible for this study.

### Procedures

Ninety patients will be recruited in the next 1.5 years based on the calculation of the authors. Baseline assessments will be completed when the participants visit the neurology clinic of Shanghai Fourth People's Hospital or Tongji Hospital. The first PWI will be completed within 24 h after onset, and the subsequent PWI will be completed on the 7th day ± 2. Three visits to the hospital are required, which include the first visit for randomization (baseline) and the next two for repeat PWI on day 7 ± 2 and follow-up on 90 ± 7 days. The incidence of recurrent vascular events will be collected from all the patients on the second and third visits. Meanwhile, etiological subtypes (large artery occlusive disease, small vessel disease, cardioembolism, etc.) should also be considered for reasons of early recurrent stroke (Purroy et al., 2007). Baseline and follow-up assessments are summarized in Table 1.

**Table 1:** Trial schedule.

**Assessment**	**Screening (within 24 h of symptoms onset)**	**Visit 1**	**Visit 2**	**Visit 3**
		**Baseline**	**Day 7 ± 2**	**Day 90 ± 7**
Demographic characteristics	×			
ABCD2 score (for TIA)	×			
Modified Rankin Scale	×		×	×
Focused medical history	×^a^			
Current medications	×^b^		×	×
MRI scan	×			
MRI PWI		×	×	×
Laboratory tests	×			
Electrocardiograph (ECG)	×			
Inclusion/Exclusion criteria	×			
Signed informed consent	×			
AEs/SAEs^c^		×	×	×
Incidence of recurrence			×	×

*AE, adverse event; SAE, serious adverse event; TIA, transient ischemic attack; ABCD2, a risk assessment tool designed to improve the prediction of short-term stroke risk after transient ischemic attacks*.

a *Relevant existing data from admission to hospital will be reviewed and recorded into the CRF. Included are concomitant diseases and medical history*.

b *The medications used during the trial should be recorded*.

c *SAEs will be recorded from the time of informed consent. AEs of interest and endpoints will be collected from the time of admission to the hospital*.

### MRI Protocol

MRI is performed using a 1.5- or 3.0-T Avanto scanner (Siemens, Erlangen, Germany). The imaging protocol consists of DWI, PWI, FLAIR, and MRA. Imaging parameters are listed below. Axial EPI-DWI: TR = 3,600 ms, TE = 102 ms; FOV = 2.3 cm^2^, *b* value = 0 and 1,000 s/mm^2^; EPI factor = 192; matrix size = 192 × 192; bandwidth = 964 Hz/pixel; 19 slices, slice thickness = 5.5 mm. Axial FLAIR: TR = 4,000 ms, TE = 92 ms; FOV = 2.3 cm^2^; TI = 1532.6 ms; matrix size = 256 × 190; bandwidth = 190 Hz/Px; 18 slices, slice thickness = 5.5 mm. Axial EPI-PWI: TR = 1,590, TE = 32 ms; measurements = 50; FOV = 2.3 cm^2^; matrix size = 128 × 128; band width = 1,346 Hz/pixel; 19 slices, slice thickness = 5.5 mm. Gd-DTPA contrast agent (gadopentetate dimeglumine; Shanghai Pharmaceutical Corporation, Shanghai, China) is intravenously injected (0.2 mmol/kg body weight) at a rate of 4 ml/s after flushing with 30 ml saline. Time-of-flight MRA: TR = 25 ms, TE = 7 ms; FOV = 1.8 cm^2^; matrix size = 241 × 256; bandwidth = 100 Hz/PX; slice thickness =0.7 mm. Ischemic lesion was localized by taking clinical manifestations of patients with TIA into consideration.

Estimates of the volume of hypoperfusion on MRI perfusion scans are performed using the RAPID software, which is an automated image post-processing software (Rothwell et al., 2007; Wang et al., 2019a). Lesion volume of *T*_*max*_ ≥ 4 s ≥ 10 ml is used for determining perfusion deficits in patients with TIA (Liu et al., 2007; Najib et al., 2019). Two experienced radiologists independently evaluated the presence of acute ischemic lesions on DWI/PWI.

### Sample Size and Power Calculation

Two-sided chi-square test is performed at *P* = 0.05 (alpha) to compare the incidence of recurrent vascular events between patients with TIA with PWI-positive and PWI-negative findings. Previous studies have shown that 10% of patients with TIA will relapse; however, the pilot study showed that 34.8% of PWI-positive patients with TIA had recurrent TIA. With the testing power of 80%, the significance level of 0.05 (two-sided), and a 1:1 ratio, with a dropout rate of 5%, a total of 90 patients are needed to detect the difference in relative risks between patients with TIA with PWI negative and PWI positive findings.

### Statistical Analysis

The proportion of patients with recurrent vascular events on days 7 and 90 will be presented as frequency (percentage). Continuous variables will be presented as mean ± SD or median with interquartile range (IQR). Categorical parameters are presented as independent proportions. Baseline information of patients with or without hypoperfusion abnormalities is compared using the Mann-Whitney *U*-test or *t*-test for continuous variables and Fisher's exact test or χ^2^ for categorical variables. Logistic regression analysis will be performed to compare the proportion of recurrence between the two groups. *P* < 0.05 (two-sided) is considered statistically significant. Data analysis will be performed using IBM SPSS (version 20.0) for Windows (SPSS Inc., Chicago, IL, USA). The Data and Safety Monitoring Board will conduct an interim analysis on 45 patients (half of the required sample size). *P* < 0.05 is considered statistically significant in the interim and final analyses.

## Discussion

Based on a multi-centered community epidemiological study on TIA in Chinese adults, the prevalence of TIA in the Chinese population is as high as 2.4%. The number of patients with TIA in China is as high as 10–12 million (Liu et al., 2007). MRI-based hypoperfusion in patients with TIA is closely related to the time of completing MR imaging. A recent study has shown that hypoperfusion, detected by MRI, is present in 42.4% (25/59) of patients with TIA, and the median (IQR) time of symptom onset to baseline MR-PWI was 5 (4–9) days (Wang et al., 2019a). This is similar to the results of a previous study that has demonstrated the presence of hypoperfusion in 42% (57/137) of patients with TIA, with a median time of 9.2 h from symptom onset to completing MR-PWI (Asdaghi et al., 2013). When the time from symptom onset to the first PWI was shortened, such as a median time of 287 (136–591) min, the presence of hypoperfusion decreased to 25% (16/64) (Lee et al., 2017).

A recent study has shown an incidence of 7.4% of 90-day recurrence of stroke after TIA (Najib et al., 2019). There are multiple potential clinical risk factors for MR perfusion abnormality in the context of TIA. Insufficient local tissue perfusion may be the cause of recurrent TIA (Wang et al., 2019a,b).

This study has its own limitations. First, all the patients were recruited from the same city (Shanghai), and there may be bias in-patient selection. Second, the number of participants is relatively small. Third, treatments for patients with TIA might be slightly different, which will influence the likelihood of TIA recurrence.

The REATTACK trial will provide the power of predicting recurrent vascular events on days 7 and 90 based on hypoperfusion observed on PWI, which is completed within 24 h after symptom onset. This will guide the implementation of effective therapeutics for secondary prevention of TIA and/or stroke.

## Ethics Statement

The studies involving human participants were reviewed and approved by Human Research Ethics Committee of Shanghai Fourth People's Hospital and Tongji Hospital. The patients/participants provided their written informed consent to participate in this study.

## Author Contributions

YW and HL participated in the design and coordination of the study and drafted the manuscript. LJ designed the study and revised the manuscript for important intellectual content. SW conceived and designed the study and supervised the entire process. All the authors read and approved the final manuscript.

## Conflict of Interest

The authors declare that the research was conducted in the absence of any commercial or financial relationships that could be construed as a potential conflict of interest.

## Abbreviations

CIs: confidence intervals
CTA: computed tomography angiography
DWI: diffusion-weighted imaging
FLAIR: fluid-attenuated inversion recovery
IQR: interquartile range
MRA: magnetic resonance angiography
MRI: magnetic resonance imaging
mRS: modified ranking scale
MTT: mean transit time
PWI: perfusion-weighted imaging
TIA: transient ischemic attack
*T*_*max*_: time-to-maximum of residue function
TTP: time to peak.
